# Eye Movement Correlates of Expertise in Visual Arts

**DOI:** 10.3389/fnhum.2018.00087

**Published:** 2018-03-26

**Authors:** Piotr Francuz, Iwo Zaniewski, Paweł Augustynowicz, Natalia Kopiś, Tomasz Jankowski

**Affiliations:** Department of Experimental Psychology, The John Paul II Catholic University of Lublin, Lublin, Poland

**Keywords:** expertise in visual arts, painting, balanced composition, aesthetic judgment, eye movement, familiarity of painting

## Abstract

The aim of this study was to search for oculomotor correlates of expertise in visual arts, in particular with regard to paintings. Achieving this goal was possible by gathering data on eye movements of two groups of participants: experts and non-experts in visual arts who viewed and appreciated the aesthetics of paintings. In particular, we were interested in whether visual arts experts more accurately recognize a balanced composition in one of the two paintings being compared simultaneously, and whether people who correctly recognize harmonious paintings are characterized by a different visual scanning strategy than those who do not recognize them. For the purposes of this study, 25 paintings with an almost ideal balanced composition have been chosen. Some of these paintings are masterpieces of the world cultural heritage, and some of them are unknown. Using Photoshop, the artist developed three additional versions of each of these paintings, differing from the original in the degree of destruction of its harmonious composition: slight, moderate, or significant. The task of the participants was to look at all versions of the same painting in pairs (including the original) and decide which of them looked more pleasing. The study involved 23 experts in art, students of art history, art education or the Academy of Fine Arts, and 19 non-experts, students in the social sciences and the humanities. The experimental manipulation of comparing pairs of paintings, whose composition is at different levels of harmony, has proved to be an effective tool for differentiating people because of their ability to distinguish paintings with balanced composition from an unbalanced one. It turned out that this ability only partly coincides with expertise understood as the effect of education in the field of visual arts. We also found that the eye movements of people who more accurately appreciated paintings with balanced composition differ from those who more liked their altered versions due to dwell time, first and average fixation duration and number of fixations. The familiarity of paintings turned out to be the factor significantly affects both the aesthetic evaluation of paintings and eye movement.

## Introduction

Aesthetic experience is a result of many factors accompanying the appreciation of a work of art. Two of them seem particularly important: the quality (aesthetic value) of the work of art and the level of expertise of the beholder (Wölfflin, 1950; Gombrich, 1995).

In this study, paintings are of interest. A reflection on the relationship between the aesthetic value of the painting and the level of the beholder’s expertise reveals that they are largely dependent on each other. On the one hand, only an expert can make an accurate and reliable assessment of the value of the painting. On the other hand, the measure of expertise is the ability to differentiate paintings because of their quality. This means that determining whether a person is an expert in visual arts can only be made on the basis of judgments of the paintings being viewed. If he or she states that the painting is valuable, and it really is valuable, then according to the classical theory of truth, such a person can be called an expert. However, to assess the expertise level of the beholder, we would have to know the actual aesthetic value of the given paintings, i.e., know who or what the criterion is of that value. As Ericsson (2014) notes, it is possible to measure the level of expertise of individuals only with tasks with well-known correct answers.

In conclusion, it seems that without the initial assumptions concerning the value of the work of art or the competence of the beholder, estimating the level of expertise in art is unsolvable. If there are no objective criteria for evaluating the quality of paintings, then we are all experts, and naming some works of art as masterpieces is merely a manifestation of someone’s subjective preferences. On the other hand, if there are criteria for assessing the aesthetic value of a painting, the first question is, What are they? Secondly, What are the indicators of effective and accurate use of them by the beholder? Specifically, the question is: Apart from someone’s claims that the given painting is outstanding (or not) can we use the more objective methods to verify his or her competence?

The answer to the question about the criteria of appreciation of paintings is suggested by the Pythagorean-Platonic tradition, pointing to a harmonious (balanced) composition of a work of art as one of the most important conditions of its aesthetic value (Tatarkiewicz, 1980). That is why, by selecting reproductions of paintings for empirical research in the field of aesthetics, some researchers are guided by the recognition of their high aesthetic value due to their compositional values, especially harmony or dynamic balance (McManus et al., 1985; Locher and Nodine, 1989; Nodine et al., 1993; Locher, 1996, 2014b; Locher et al., 2005; Vartanian et al., 2005; Wilson and Chatterjee, 2005; Gershoni and Hochstein, 2011; McManus et al., 2011; Jahanian et al., 2015; Abeln et al., 2016).

From the statements of outstanding artists, for whom the composition is a special challenge, it follows that the harmonious composition of the painting is by no means merely a simple result of proportionally or symmetrically arranged elements on the surface of the painting. However, in accord with the Matisse idea contained in *Notes d’un peintre* (1908), “In a picture every part will be visible and will play its appointed role, whether it be principal or secondary. Everything that is not useful in the picture is, it follows, harmful. A work of art must be harmonious in its entirety” (Flam, 1978, p. 36). Bradley (2013) adds that “when every element in your work agrees with every other element harmony is achieved” (p. 174). An important aspect of a balanced composition is also that it “refers to the way in which disparate elements of an image produce visual forces that compensate for each other… in which unity can be rendered in diversity” (Wilson and Chatterjee, 2005, p. 166).

The quotes explicitly suggest that the painting elements are in harmony if and only if they are distributed in the painting space so that they simultaneously balance the intensity of all the features. A violation of any proportion between elements—that is, by adding, shifting, or deleting an element, or modifying the intensity of one—implies a violation of the whole composition (Crozier and Chapman, 1984). The arrangement of these elements can be recognized only by shifting the gaze from place to place in a specific order and time. Thus, the registration of eye movement provides the most primary information on potential differences between a harmonious and non-harmonious painting composition as well as their aesthetics.

The interference in a harmonious painting cannot be either mechanical (e.g., by imposing theoretically unjustified filters with Photoshop) or schematic (e.g., by shifting or deleting certain elements of the painting to obtain a more symmetrical or proportional arrangement of them relative to the selected axis of symmetry). On the contrary, first, the interference in the composition of the painting should be made by the artist with a very high sense of the relationship between elements of the composition. Second, the artist should have full freedom in choosing the method of interference in the composition, constantly checking the degree to which it is violated. Third, the changes should not look like foreign structures on the background of the original. This technique of modifying composition of paintings has been used in our study by painter and photographer, Iwo Zaniewski.

The second factor influencing the appreciation of the work of art is the level of expertise of the observer. Ericsson (2014) claims, “When someone has gained special skills or knowledge representing mastery of a particular subject through experience and instruction, we call this person an expert” (p. 508), and Ericsson and Lehmann (1996) define expert performance as “consistently superior performance on a specified set of representative tasks for a domain” (p. 277). In both definitions, attention is drawn to two aspects of expertise, understood as “gained special skills or knowledge” or “superior performance on a set of tasks”. Considering the first definition, an expert is a person who has acquired skills or knowledge, such as through education or training, and considering the second one, an expert is a person who solves a certain class of problems better than others. In any case, an expert is expected to make the right decisions, solve problems, and answer the most important questions. It seems that from an expert in art we can expect a reliable answer to the question of the quality of an artistic work. Specifically, if we agree that a given painting has a harmonious composition and if the artist violates it, then we can expect that by evaluating both versions of this image, the expert in visual art will indicate the unchanged (original) version as more valuable than the changed one.

In the field of empirical aesthetics, students of the history of art and fine arts are most often recruited for research as experts (Kristjanson and Antes, 1989; Hekkert and van Wieringen, 1996b; Augustin and Leder, 2006; Pihko et al., 2011; Leder et al., 2013; Koide et al., 2015). Artists with different experiences and accomplishments (Winston and Cupchik, 1992; Hekkert and van Wieringen, 1996a; Vogt and Magnussen, 2005, 2007), people who declare interest in art in specially designed questionnaires for this purpose (Belke et al., 2006; Brieber et al., 2014), or get high marks in artistic skills tests (Kozbelt, 2001) participate much less frequently in this kind of research as experts.

The results of comparative studies between experts and non-experts in the field of visual arts, reveal differences in terms of aesthetic evaluations to viewed paintings. For example, experts appreciate original paintings more than altered versions (Hekkert and van Wieringen, 1996a), and are more sensitive to the composition of the painting than non-experts (Nodine et al., 1993; Locher et al., 1999, 2005). The differences between experts and non-experts in the field of visual arts are also found in eye tracking studies. It turns out that when viewing and/or assessing the aesthetic value of paintings, the oculomotor activity of experts compared to non-experts is characterized by longer saccades (Zangemeister et al., 1995; Kapoula and Lestocart, 2006; Vogt and Magnussen, 2007; Pihko et al., 2011), and less frequent and shorter fixations on narrative elements (Kristjanson and Antes, 1989; Nodine et al., 1993; Vogt and Magnussen, 2007; Pihko et al., 2011) and salient areas (Koide et al., 2015). Locher et al. (2015) found that the time of the first fixation on paintings is longer in the experts, independent of their alleged authenticity status conditions: originals, copies, or fakes. It is also noted that the experts lead to longer and fewer fixations on paintings than non-experts (Kristjanson and Antes, 1989; Nodine et al., 1993), although McSorley and McCloy (2011) claim that this trend also applies to non-experts. Reducing the number of longer fixations in experts than non-experts is interpreted as an indicator of greater efficiency in extracting information from the fixation area. Although in the cited studies the presentation of the paintings was sequential, it can, nevertheless, be expected that this study will also have similar effects in the group of experts, especially when viewing paintings with a more harmonious composition.

Three issues should be clarified: (1) familiarity of paintings; (2) how they are presented; and (3) measuring their aesthetic value.

(1) An important feature of the painting is the extent to which it is known. Recognition of a painting as familiar (e.g., because of education) may cause the observer to appreciate its aesthetic value, not because of its great composition or originality, but because he has already seen it. The results of the research on the effect of mere exposure indicate that there is a significant relationship between the frequency of viewing a painting and its appreciation (Bornstein, 1989; Cutting, 2003). Cupchik and Gebotys (1990) found that unfamiliar paintings are evaluated as more interesting than familiar ones. In turn, Leder et al. (2013) stated that experts in visual arts showed higher emotions to more familiar paintings than unfamiliar. Kristjanson and Antes (1989) and Antes and Kristjanson (1991) identified two opposing patterns of viewing known and unknown paintings by artists and non-artists. The artists had a higher density of fixation and a shorter average duration of fixation on noncenters of unknown paintings and a lower density of fixations and a longer average duration of fixation on noncenters of known paintings. The pattern of eye movement by non-artists was the opposite. To reduce the effect of individual differences on the aesthetic evaluation of paintings, in some studies give up the exposure of known paintings at all (e.g., Cela-Conde et al., 2009; Pang et al., 2013; Cattaneo et al., 2017).

Controlling the level of familiarity of paintings in experiments in which their aesthetic value is measured is important, although it raises some methodological difficulties. The easiest way is to directly ask the observer if he has ever seen a given painting. Regardless of whether the question about the familiarity of the painting will be asked before or after asking about its aesthetic value, we can always expect the priming effect.

Another method is to collect data on the familiarity of a given painting from an independent sample from the same population as participants in the experiment in which its aesthetic evaluation is carried out. This technique solves the problem of priming effect but raises doubts about the accuracy of estimating the level of knowledge of paintings by participants in the aesthetic experiment. Finally, experts in visual arts can choose paintings of unquestionable fame, recognizing them as familiar and paintings by an unknown artist, recognizing them as unfamiliar. In our research, we combined methods for the selection of known and unknown paintings by experts and estimating their familiarity by an independent sample (results of this study see below in the section “Stimuli”).

(2) In the vast majority of research in the field of empirical aesthetics, the method of sequentially displaying individual paintings in random order is used. This procedure is used even in these studies, aimed at comparing two versions of the same painting, for example, the original and altered, or one that is like another due to a feature such as style or artist (Nodine, 1982; Kristjanson and Antes, 1989; Nodine et al., 1993; Hekkert and van Wieringen, 1996a; Locher et al., 1999; Vartanian and Goel, 2004; Pinto et al., 2006; Swami et al., 2007; Calvo-Merino et al., 2010). In some studies, two, three, or even four paintings are presented simultaneously, and the task of the participants is to select the one that best meets the criterion suggested in the instructions (e.g., aesthetic value) or order them according to this criterion (Child, 1965; Noll, 1966; Cupchik, 1974; Winner et al., 1987; Cupchik and Gebotys, 1988; Cupchik et al., 1992; McManus et al., 1993; Furnham and Rao, 2002; Cutting, 2003; Locher, 2003; Vartanian et al., 2005; Augustin et al., 2008; Arielli, 2012; Belke et al., 2015). Sometimes, a larger collection of paintings or photographs is presented at the same time, to be categorized (Augustin and Leder, 2006) or for aesthetic evaluation (McSorley and McCloy, 2011).

It seems that the method of simultaneous presentation of several paintings, compared to each other by reference to the selection criterion specified in the instruction, is most justified to identify expertise competencies. As Furnham and Rao (2002) point out, in the task of comparing paintings presented simultaneously, the cognitive processes that focus on their good/bad qualities are activated, while the evaluation of sequentially presented paintings is more determined by social factors and more often associated with liking. An additional limitation of the sequential method of presentation and comparing a pair of paintings is that their evaluation by a given criterion must be based on memory for at least one of them. It is also worth noting that the presence of multiple images at the same time in the field of view of the beholder seems more akin to a natural one (e.g., in a museum, a magazine, the Internet) than focusing on a single painting isolated from the context of other images.

(3) This note concerns the method of assessing the aesthetic value of the presented paintings due to their balanced composition. In experiments, either a direct or indirect form of the question about the aesthetic value of the composition is used. Sometimes both are used simultaneously. The first group includes sentences such as, “Which one in the pair is more (or less) harmonious? Which one in the pair contains more (or fewer) errors and faults in its configuration? (left/right)” (Götz et al., 1979; see also e.g., Locher, 2003; Vartanian et al., 2005; Wilson and Chatterjee, 2005; Winner et al., 1987). An example of an instruction belonging to the second group is, “Which picture is more strongly liked or preferred (A or B)?” (Noll, 1966; see also e.g., McManus et al., 1993; Wilson and Chatterjee, 2005).

In this study we used instructions that do not directly ask about the aesthetic value of a work of art: “Choose the version that you judge to be more pleasant while viewing (left/right)”. This form of instruction was used for two reasons. First, in addition to the experts, in the experiment naive participants took part who were not interested in visual art, and phrases such as “better,” “harmonious,” or “balanced” composition, or “error in the configuration of elements,” may be incomprehensible or ambiguous. Second, in works on the theory and history of art and aesthetics, it has been repeatedly emphasized that works of art with a balanced composition look more pleasing than those of a composition that is distorted (Wölfflin, 1950; Bouleau, 1963; Poore, 1967; Tatarkiewicz, 1980; Gombrich, 1995; Roberts, 2008; Bradley, 2013). The evaluation of paintings as more or less pleasing to the eye is free from the social and cultural context (unlike, for example, the “liking”) and from the context of knowledge (as in evaluating paintings due to the level of balance of their composition).

Inspired by the results of the presented research, we asked the following questions: Do experts and non-experts in the field of visual arts differ in terms of aesthetic evaluation of original paintings with a perfectly balanced composition and their altered versions with an unbalanced composition? In other words, to what extent does education in the field of visual arts correlate with the aesthetic evaluation of harmonious and disharmonious paintings? To what extent does the familiarity of these paintings affect their aesthetic evaluation? Does the eye movement of people who value paintings with harmonious compositions more than non-harmonious ones differ from those of people who prefer paintings with a non-harmonious composition rather than a harmonious one? Does the familiarity of the viewed paintings affect their eye movements?

Based on the results of experiments, in which the task of the subjects was to compare original paintings (most often with balanced compositions) and their altered versions (e.g., Noll, 1966; Nodine, 1982; Neperud and Marschalek, 1988; McManus et al., 1993; Nodine et al., 1993; Locher and Nodine, 1994; Hekkert, 1995; Hekkert and van Wieringen, 1996b; Locher et al., 1996, 1999; Vartanian et al., 2000, 2005; Furnham and Rao, 2002; Locher, 2003, 2016; Vartanian and Goel, 2004; Swami et al., 2007; Pihko et al., 2011; Pang et al., 2013; Koide et al., 2015) we expected that: (1) experts, compared to non-experts, will have a greater appreciation of the aesthetic value of original paintings than their altered versions; and (2) in the group of experts, this effect will be partially independent of the familiarity of the paintings because the choice of a familiar original painting as more aesthetically valuable than its altered version may also be a result of its recognition. This means that although experts should more accurately assess the aesthetic values of both known and unknown paintings than non-experts, this effect should be stronger in the group of known than unknown paintings, independently of the level of expertise.

To the best of our knowledge, in only a few studies, the task of the subjects was to compare the aesthetic values of the paintings exhibited simultaneously in pairs using eye tracker (e.g., Locher and Nodine, 1994), but even in this study, only the unsophisticated group in the visual arts participated. Therefore, predicting differences between experts and non-experts, as well as between the eye movement strategies of viewing different versions of paintings is greatly limited. Considering the research results, it seems that the correlation between different measures of eye movement and expertise is by no means unambiguous. We only expect that experts and people who accurately assess the composition of paintings use other oculomotor strategies than non-experts and people who can not do that. Searching for oculomotor markers of expertise, according to the paradigm proposed in this study is, therefore, largely exploratory.

## Materials and Methods

### Participants

The study involved 23 experts in art (students of art history, art education, or the Academy of Fine Arts), aged 23.8 years on average, including 18 females, and 19 non-experts (students in the social sciences and the humanities), aged 23.7 years on average, including 14 females. All participants had normal or corrected to normal vision. They were paid for their participation in the study of approximately $25.

### Stimuli

Digitized reproductions of 25 figurative paintings, 16 masterpieces of world painting by famous artists, and nine by Iwo Zaniewski (a relatively unknown Polish painter and photographer) were used as a stimulus. The common feature of all these paintings is almost perfectly balanced composition, so any modification of such paintings could only destroy their harmony. Among the masterpieces were such paintings, as *Joy of Life* by Henri Matisse, *The Young Ladies of Avignon* by Pablo Picasso, *Bathers at Asnières* by George Seurat, or *Virgin and Child Surrounded by Angels* by Jean Fouquet (see Supplementary Material). The paintings were selected by a group of art historians and artists with more than 30 years of experience in the profession.

To ensure that the paintings by famous artists are more familiar than paintings by an unknown artist, an additional test of their familiarity on an independent sample (*N* = 167, including 35 experts) was carried out. As a result of the study, it turned out that some of the paintings by famous artists are, in fact, unknown to both experts and non-experts. Therefore, only nine of them (out of 16) were considered for further analysis.

**Figure 1:**
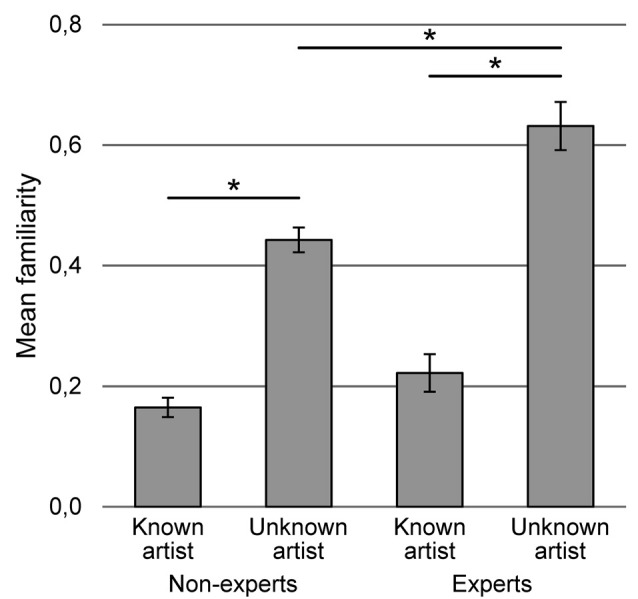
The interaction of variables expertise and artist on familiarity of the painting. Vertical bars denote ± standard errors, *means a significant difference.

A two-way ANOVA with repeated measures of variable artist (known, unknown) and inter-group variable expertise (expert, non-expert) was performed. According to our expectations the main effects of artist (*F*_(1,165)_ = 280.632, *p* < 0.001, *η*^2^ = 0.630) and expertise (*F*_(1,165)_ = 12.570, *p* = 0.001, *η*^2^ = 0.071) were found. Paintings by known artist were more familiar than the paintings by unknown artist and experts were more familiar with both groups of paintings than non-experts. The interaction between expertise and artist (*F*_(1,165)_ = 10.331, *p* = 0.002, *η*^2^ = 0.059, Figure 1) was also found. Bonferroni-corrected *post hoc* tests revealed differences between familiarity of the paintings by known and unknown artists both in the group of experts (*p* < 0.001) and non-experts (*p* < 0.001) and between experts and non-experts in the relation to the paintings by known artists (*p* < 0.001). The difference between experts and non-experts in relation to the paintings by unknown artist was insignificant.

**Figure 2:**
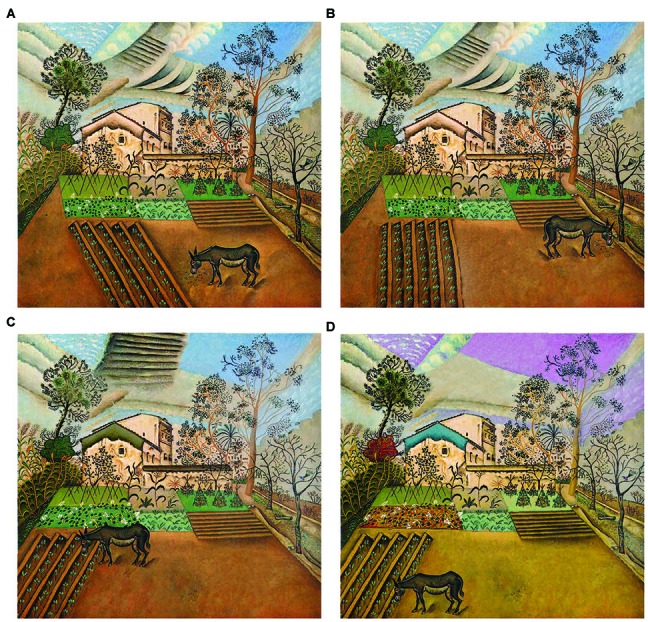
**(A)** Joan Miro, *The Vegetable Garden with Donkey*, 1918, oil on canvas, 70 × 64 cm, Moderna Museet, Stockholm, Sweden, **(B)** slightly modified version of **(A)**, **(C)** moderately modified version of **(A)**, **(D)** significantly modified version of **(A)**.

Every original painting (marked with the letter A) was modified in Photoshop to a slight (B), moderate (C), and significant (D) degree. The changes concerned brightness, saturation, color, location, and/or size of some elements on the paintings. The purpose of the painting modification was to disrupt their balanced composition to varying degrees. The decision to modify the paintings at a given level (B, C, or D) was based on the intuitive scale of the painting composition’s destruction, accepted by the artist (Iwo Zaniewski) performing the task (Figure 2). It was assumed that the modification of the paintings’ composition would negatively affect their aesthetic evaluation in particular by experts with a greater visual literacy. To test this assumption, the evaluations of all AB, AC, and AD pairs of paintings made by visual arts experts and non-experts were compared (see “Results” section).

### Apparatus

The paintings were displayed on a computer screen with a resolution of 1920 × 1200 pixel. The SMI (SensoMotoric Instruments GmbH) Iview-X HiSpeed 1250 (sampling rate of 1250 Hz, latencies of less than 0.5 ms, high accuracy: 0.25°–0.5°) eye tracker was used to record eye movement. A dispersion-based fixation detection algorithm was used with the following parameters: minimum fixation duration = 80 ms, maximum dispersion = 100 px. The program for exposing paintings and registering reactions of participants was written using E-Prime 2.0. The subjects sat about 70 cm away from the screen and answered using the keyboard with three buttons.

### Procedure

At the beginning of the experiment, each participant was informed that the aim of the study was to seek answers to the question of why some paintings are more pleasant to view than others and that there are no right or wrong answers but more importantly, that they are sincere and express their personal feelings. Following this, they viewed four paintings simultaneously (original and its three altered versions), located in random order on the screen and were asked to look at each of them and try to notice all the differences. The participants had as much time as they wanted to look at all the paintings. In the next phase, they saw four versions of a painting presented separately, also in random order. This phase has been introduced only to allow participants to view all paintings versions more accurately (one painting on the screen). At the end, in the third phase of the study, all versions of the picture were twice presented in pairs and participants had to choose the version that they judged as more pleasant. Participants had as much time as they needed for their decision. The duration of the experiment was from 1 h to 3 h depending on the participant (*M* = 132.49 min, SD = 53.52 min). The participant could ask for a short break at any time during the experiment.

The order of the paintings’ presentation in pairs was fully randomized. The same version of a painting was presented on the left and on the right side of the screen the same number of times. This procedure was repeated for all sets of four paintings. The sequence of the presentation of the sets of paintings was random. During all phases of the experiment, the participants’ eye movements were recorded. Behavioral and oculomotor data recorded during the first and the third phase of the experiment were analyzed statistically.

### Independent and Dependent Variables

The following independent variables were considered in different analyses: (1) expertise: experts (students of art history, art education, or the academy of fine arts), non-experts in art (students in the social sciences and the humanities); (2) familiarity of the painting (known, unknown); and (3) difference between paintings in pair: slight (pair AB), moderate (pair AC), significant (pair AD). Only three (out of six) pairs of paintings were considered for statistical analysis. In each of these pairs was the original painting, i.e., A and one of its altered versions (B, C, or D).

The following dependent variables were considered: (1) accuracy of paintings evaluation in pair: 0 means a not-apt indication of the painting modified as being better than the original, and 1 means an apt indication of the original painting as being better than the modified; (2) dwell time (ms) is the sum of durations all fixations and saccades inside one of the painting from a pair during evaluation of paintings; (3) average fixation duration (ms) is the sum of fixations time divided by the number of fixations inside a painting AOI; (4) first fixation duration (ms) is the duration of the first fixation on the painting; (5) fixation count (Qty) is the sum of all fixations inside a painting. All these variables are included in the analysis, although some of them are correlated with each other, e.g., dwell time and the number of fixations, because the results of statistical analyses reveal other effects (see below).

## Results

### Accuracy of Paintings Evaluation in Pairs

We used R (R Development Core Team, 2008), as well as lme4 (Bates et al., 2015), lsmeans (Lenth, 2016), car (Fox and Weisberg, 2011) and MuMln (Barton, 2016) packages to perform a generalized linear mixed effects analysis. As fixed effects, we entered the difference between paintings in a pair (slight: pair AB; moderate: pair AC; and significant: pair AD), familiarity of a painting (known, unknown) and inter-group variable expertise (expert, non-expert) as well as all possible interaction terms between predictors into the model. Individual observations on the level-1 are cross-classified at level-2 by both participants and paintings, therefore as random effects, we entered intercepts for subjects and painting, as well as by-subject random slopes for the effect of difference between paintings in pair. The whole model explained 23% of variance of dependent variable (with 11% of variance explained by fixed effects and 12% explained by random effects). The *p* values were adjusted with Tukey method for comparing simple effects.

**Figure 3:**
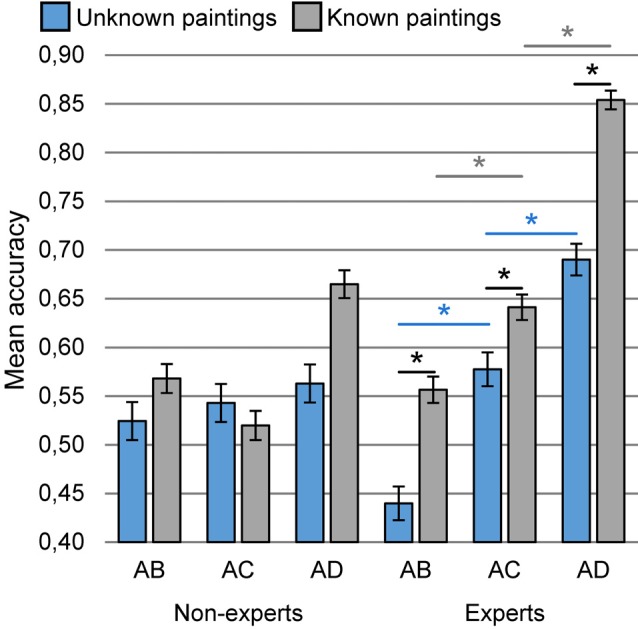
The interaction of expertise, familiarity and difference between paintings in pair on accuracy of paintings evaluation during evaluation of two versions of the same painting in three pairs: AB, AC and AD. Vertical bars denote ± standard errors, *means a significant difference.

The analysis of deviance of evaluation accuracy (type III Wald chi square tests) revealed a significant interaction of all three variables: expertise, familiarity and difference between paintings in pairs (*χ*^2^ = 5.90, *df* = 2, *p* < 0.052, Figure 3). The presence of the variable expertise in the interaction with variable familiarity and difference between paintings in pairs reveals that only in the group of experts, the greater the degree of violation of the harmonious composition of paintings, both known and unknown, the greater is the accuracy of the assessment of their value. Differences in the accuracy of the assessment of known paintings between the AB and AC, AB and AD pairs as well as AC and AD in the expert group were significant (*t* = −4.12, *p* < 0.002, *t* = −8.51, *p* < 0.001 and *t* = −6.56, *p* < 0.001, respectively). Similarly, differences in the accuracy of unknown paintings between pairs AB and AC, AB and AD, and AC and AD in this group were significant. (*t* = −4.59, *p* < 0.001, *t* = −5.66, *p* < 0.001 and *t* = −3.17, *p* < 0.066, respectively). On the other hand, in the group of non-experts, there were no differences in the accuracy of assessments between successive pairs of paintings, both known and unknown. In the group of experts, there were also significant differences between the accuracy of the assessment of known and unknown paintings in individual pairs AB, AC and AD (*t* = −3.57, *p* < 0.038, *t* = −3.07, *p* < 0.089 and *t* = −6.50, *p* < 0.001, respectively), whereas in the non-expert group such differences were not found.

### Eye Movement Preceding the Appreciation of Paintings

To analyze data from viewing pairs of versions of the same painting presented simultaneously, we estimated four models with the following dependent variables, i.e., dwell time, average fixation duration, first fixation duration and fixation count. We entered familiarity of painting (known, unknown), difference between paintings in pair (slight: pair AB; moderate: pair AC; and significant: pair AD), accuracy of paintings evaluation in pair (0, 1) and the intergroup variable, expertise (expert, non-expert) into models as fixed effects. As random effects, we entered intercepts for subjects and paintings, as well as by-subject random slopes for the effects of difference between paintings in pair and accuracy of paintings evaluation. In the case of dependent variables based on time measurement we performed linear mixed effect analysis, however the values of the oculomotor variables were subjected to logarithmic transformation in order to normalize their distribution. In the case of fixation count variable, we conducted generalized linear mixed effects analysis assuming the Poisson distribution of these dependent variables.

The variable accuracy of paintings evaluation in pair was included in the analysis of oculomotor data as an independent variable, based on behavioral data. In both groups (experts and non-experts), 64% of the participants accurately appreciated painting A as more valuable than its altered version in all comparisons in pairs and 36%, conversely. However, it turned out that, on the one hand, some experts evaluated the modified versions of paintings higher than the original, and, on the other hand, some non-experts pointed to the original paintings as more valuable than their altered versions. Consequently, in this analysis, two dimensions of expertise were considered: nominal and executive. The first is the membership in a group of experts or non-experts, based on education (variable: expertise). The second one is the accurate evaluation of a painting that occurred at the particular trial (variable: accuracy of paintings evaluation in pair). This distinction directly refers to the division of experts because of education or training, and experts because of their high ability to solve a certain class of problems better than other people (Ericsson and Lehmann, 1996; Ericsson, 2014). In other words, we were interested how expertise understood as a personal characteristic (variable on the participant level) is related to oculomotor behavior compared to expertise defined as an ability to distinguish more valuable version of a painting in a pair (variable on the observation level).

#### Dwell Time

The significant interaction between accuracy of paintings evaluation and familiarity (*χ*^2^ = 16.29, *df* = 1, *p* < 0.001) was found. With reference to known paintings, it was found that the shorter the dwell time, the higher the accuracy of paintings evaluation (*t* = 4.47, *p* < 0.001). A similar, though statistically insignificant tendency was also found in relation to unknown paintings (*t* = 2.22, *p* = 0.129). There were also no differences between known and unknown paintings, whose aesthetic value was both accurately and inaccurately evaluated. The main effect of difference between paintings in pair (*χ*^2^ = 98.96, *df* = 2, *p* < 0.001) was also found to be significant. When comparing paintings in pair AB, dwell time was significantly longer than when comparing paintings in pairs AC (*t* = 12.78, *p* < 0.001) and AD (*t* = 16.83, *p* < 0.001), and when comparing paintings in pair AC than AD (*t* = 6.91, *p* < 0.001). There were no main or interaction effects involving expertise. The model explained 36% of variance of dwell time (with 13% related to fixed effects).

#### Average Fixation Duration

Two main effects of accuracy of paintings evaluation (*χ*^2^ = 4.44, *df* = 1, *p* < 0.035) and familiarity (*χ*^2^ = 4.79, *df* = 1, *p* < 0.029) were found. If the average fixation duration was longer when comparing two paintings, then the original one was less frequently indicated as better than its altered version. The average fixation duration was also longer when viewing known paintings than unknown. No other significant effects were found. The model explained 21% of variance of average fixation duration (with only about 1% related to fixed effects).

#### First Fixation Duration

**Figure 4:**
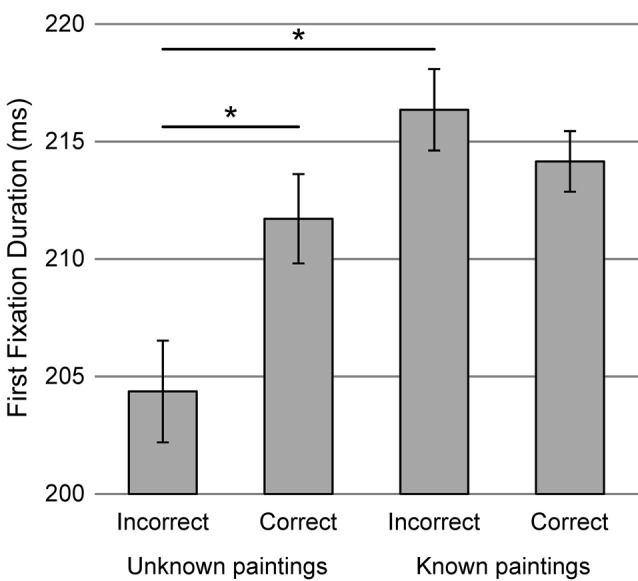
The interaction of familiarity and accuracy of paintings evaluation in pair on the first fixation duration during evaluation of two versions of the same painting. Vertical bars denote ± standard errors, *means a significant difference.

The interaction effect of familiarity and accuracy of paintings evaluation in pair (*χ*^2^ = 11.27, *df* = 1, *p* < 0.001, Figure 4) was found. Longer duration of the first fixation was observed on known painting compared to unknown one but only when it was subsequently evaluated inaccurately (*t* = 4.04, *p* < 0.001). When the original painting was appreciated more, familiarity did not predict this variable (*t* = 0.91, *p* = 0.798). The model explained 9% of variance of first fixation duration (with only less than 1% related to fixed effects).

#### Fixation Count

**Figure 5:**
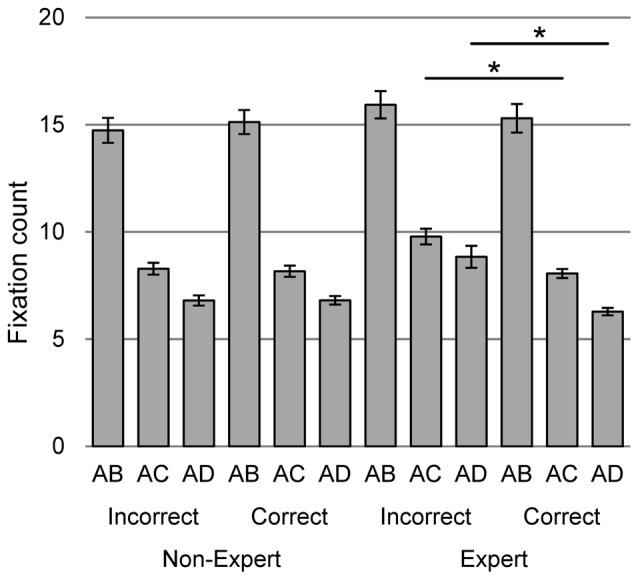
The interaction of accuracy of paintings evaluation, expertise and difference between paintings in pair on the number of fixation during evaluation of two versions of the same painting. Vertical bars denote ± standard errors, *means a significant difference.

The three-way interaction was found between accuracy of paintings evaluation, expertise, and difference between paintings in pair (*χ*^2^ = 48.33, *df* = 2, *p* < 0.001, Figure 5). This interaction reveals that the higher number of fixations predicts the lower accuracy evaluation of paintings, which are more different in pairs (i.e., AC and AD), but only in group of experts (*t* = 5.19, *p* < 0.001, *t* = 5.90, *p* < 0.001, respectively). There are no analogous differences in the non-expert group, and in relation to the pairs in which the paintings differ slightly (i.e., AB).

Familiarity interacted both with expertise (*χ*^2^ = 45.06, *df* = 1, *p* < 0.001) and accuracy of painting evaluation (*χ*^2^ = 36.77, *df* = 1, *p* < 0.001). The number of fixations on unknown paintings was much higher than on known ones but only in experts (*t* = 2.76, *p* < 0.027). Similarly to the dwell time, with respect to known paintings that were accurately evaluated, a significantly lower number of fixations were found than for the incorrectly evaluated paintings (*t* = 4.63, *p* < 0.001). The same, though insignificant, tendency was noted for unknown images. It was also found that an accurate evaluation of unknown paintings was preceded by a significantly higher number of fixations than for known images (*t* = 2.74, *p* < 0.003). The model explained 37% of variance of average fixation duration (including 15% explained by fixed effects).

## Discussion

In our research, we set two goals. The first objective was to verify the hypothesis that experts in the field of visual arts more often appreciate the aesthetic value of paintings with a balanced composition than the violated one. We expected that artistic education or in the field of art history sensitizes the perception of harmony, one of the most classic compositional principles. The second goal was to check whether there is a relationship between the eye movement that precedes the accurate and inaccurate painting assessment. In other words, we were interested in whether people who accurately evaluate a painting look at it in the same way as those who do not like it. Although the results of many studies in the field of empirical aesthetics indicate such a possibility, it is relatively rarely used a strategy of simultaneous presentation of paintings with a balanced and unbalanced composition in pairs, compared due to their aesthetic values. Therefore, we have not formulated detailed hypotheses regarding the relationship between the characteristics of the composition, the accuracy of its recognition and specific parameters of eye movement.

The accuracy of evaluations of paintings turned out to be significantly higher by the experts than non-experts and with respect to the AD pair than to AC, or AB and with respect to AC pair than to AB, but only in the expert group. The results have largely confirmed our first hypothesis. They are also consistent with the results of these experiments, where the subjects differentiated original paintings and their altered versions. For example, Hekkert and van Wieringen (1996b) found that altered versions of paintings were less liked and regarded as less balanced than the original (figurative, in this case) ones. Similarly, Nodine (1982) found that violation of a composition based on the golden section has a negative effect on the aesthetic evaluation of these paintings. He concludes that for experts in visual arts the visual structure of a painting, attention strategies and judgments of compositional design are intimately related. Also, Locher et al. (1999) stated that experts in visual arts were significantly more successful in detecting the original than the altered versions of paintings than were non-experts.

The familiarity of paintings has also proved to be an important factor affecting the accuracy of paintings evaluation with different compositions. Known, balanced paintings were more often aptly indicated as more valuable in terms of aesthetics than unknown. This difference applies to all three pairs of images to a greater or lesser degree: AB, AC and AD. However, variable familiarity of paintings is of particular importance in interacting with variables expertise and difference between paintings in pair. It turns out that experts are not only sensitive to modifications introduced to subsequent versions of both known and unknown harmonious paintings. The degree of modification of known and unknown paintings, however, does not affect the assessment of their aesthetic value by non-experts. This effect fully confirms our second hypothesis that the experts accurately recognize modifications to a harmonious composition of paintings regardless of their familiarity. If the experts were more aptly evaluating the aesthetic value of only known paintings, one would suspect that they simply remember them better. However, the reported effect clearly indicates that the greater accuracy in the assessment of paintings in the group of experts is not because they remember the original versions of so-called known paintings, but because they are really sensitive to the variable that has been manipulated in this study, i.e., a harmonious composition.

In conclusion, the paradigm of selection, development, and presentation in pairs of paintings with a balanced composition and their altered versions, used in this study, proved to be an effective tool for differentiating experts from non-experts in the field of visual arts. Students of art history and fine art more accurately recognized the harmonious composition of the paintings and were more sensitive to the degree of its destruction than non-experts. The accuracy of the assessment of unknown paintings is a measure of their sensitivity to the violation of a balanced composition. Therefore, it was examined whether and to what extent their expertise was revealed in eye movement while viewing and comparing simultaneously presented pairs of paintings, original and its altered versions.

The longest dwell time was found for pairs in which the original and modified paintings differed the least (AB pair), slightly shorter for the AC pair and the shortest for the pair AD. This effect explains the difficulty in differentiating paintings in a pair before indicating which one is more valuable in terms of aesthetics. Regarding the AB pair, this was the most difficult task, hence the dwell time was the longest. This effect is independent of the accuracy of the assessments, of the expertise, understood as the result of education, as well as of familiarity of the paintings.

Shorter dwell time was recorded while watching well-known paintings and their altered versions, which were accurately evaluated in terms of aesthetics, than when viewing well-known paintings, which were evaluated inaccurately. A similar but insignificant trend was also found for unknown paintings. This effect is independent of the observer’s education. Considering that dwell time refers to the time preceding the moment of pointing to one of the pictures in pair, as more valuable aesthetically, one can assume that it is not irrelevant to the accuracy of this decision. Perhaps the mechanism of appreciating the harmonious composition of images is the more effective the faster and less analytically (easier) it works. This interpretation is also confirmed by the result of analogical interaction with reference to the number of fixations. The more elements of the painting are analyzed visually, the more likely it is that inaccurate decisions regarding the aesthetic value of the compared paintings will be taken.

The presented results can be interpreted in the perceptual fluency theory of beauty (Reber et al., 2004). According to this theory, the perception of beauty is a resultant fluent processing of the object (e.g., painting) by perceiver and specific properties of this object, like goodness of form, symmetry, figure–ground contrast which facilitate fluent stimulus processing. Reber et al. (2004) claims that the more fluently the object is process, the more positive its aesthetic evaluation. This theory is based on an interactionist perspective in the aesthetics within which a sense of beauty emerges from patterns in the way people and objects relate. Referring this theory to the reported results of our research, recognizing a specific compositional pattern in the painting, e.g., harmony, is possible only if the observer has a mental representation of such a pattern. For those who have a pattern of a harmonious composition of elements in a painting, its discovery in one of the two compared paintings is easy or fluent, requires less time and effort, and at the same time is the source of its positive aesthetic evaluation. In turn, the lack of mental representation of the pattern of a balanced composition can make it difficult to decide which of the two paintings is more valuable in terms of aesthetics. The final indication of a painting with a less balanced composition as more valuable may be more or less accidental but certainly requires more time.

A similar effect was found with respect to the average fixation duration. It turns out that regardless of the familiarity of paintings or education in the field of visual arts, paintings aptly chosen as more attractive in terms of aesthetics, were also characterized by a shorter average fixation duration during their viewing. In eye tracking studies, a shorter average fixation duration is more often recorded during the exploration of the entire visual scene (global strategy) than during eye concentration on its fragments (local strategy; Locher and Nodine, 1989; Nodine et al., 1993). The discovery of the value of painting composition is possible by a global, rather than local, strategy. The global strategy is most often used by artists sensitive to the composition of paintings (Zangemeister et al., 1995; Locher, 1996; Locher et al., 2007). It seems, therefore, that an accurate assessment of the image in terms of its composition requires a global strategy of its viewing, which is associated with a shorter average fixation duration. In our study, this effect, although significant, is rather subtle.

We found that average fixation duration was longer when viewing pairs of known images than unknown. Kristjanson and Antes (1989) stated that the mean fixation duration located on noncenters of interest for artists viewing familiar paintings was significantly longer than while viewing unfamiliar ones, and longer in comparison to nonartists. In our research, we did not find the interaction between the familiarity of paintings, and expertise and the accuracy of paintings evaluation. Average fixation duration is interpreted as a measure of the involvement of cognitive processes in the interpretation of visual data collected at the sites of eye fixation. Thus, a longer average fixation duration when viewing known paintings could result from the need for a more intensive search of memory resources to recall the appearance of the previously viewed painting. Such memory exploration was not necessary for unknown paintings and therefore the average fixation duration when viewing them was significantly shorter.

We also found that the first fixation duration proved to be significantly related to the accuracy of paintings evaluation, but only if the painting was unknown: a longer first fixation duration was preceded only by an accurate assessment of unknown paintings. There are even fewer results regarding first fixation duration as an indicator of expertise in visual arts. Locher et al. (2015) found that participants sophisticated in art had longer first fixation times on lesser known paintings by major artists than naïve participants. However, they did not control the quality of the composition of these paintings, nevertheless, they controlled the familiarity of these paintings and regarding the first fixation duration, the results of their study are consistent with the results of our research.

In empirical aesthetics studies, the first fixation is often removed (e.g., Kristjanson and Antes, 1989). Much more often, eye movements are analyzed from the first second after onset of the exposure time of the single painting (e.g., Kapoula et al., 2008), the first five (e.g., Vogt and Magnussen, 2007), or first 10 s (e.g., Pihko et al., 2011). This idea is based on the concept of the two phases of an aesthetic experience with paintings, according to which, during the first few seconds of viewing the painting, its aesthetic value is determined, and then the viewing time is devoted to more detailed exploration (Locher et al., 2007; Locher, 2014a). Simultaneous exposure of two or more paintings causes that the number of fixations of eyes on paintings being compared do not necessarily evenly distribute on them. While it is justified to estimate the time of the first fixation on one of the compared paintings (analogously as its time it is estimated after entering the area of interest determined on a single painting), it is problematic to compare the average fixation time estimated for a predetermined time, e.g., 1 s, 5 s, or 10 s. The result of this comparison is extremely difficult to interpret, especially considering the theory of the two phases of aesthetic experience.

The effect of the difference between paintings in pair on number of fixations turned out to be analogous to that of dwell time. The difficulty of the task resulting from the differences between the images in the pairs caused that the number of fixations on paintings more like each other, i.e., in the pair AB was significantly higher than in the paintings differing more from each other, i.e., in AC or AD pairs. The interaction of the familiarity of paintings and accuracy of painting evaluation was also similar in relation to the number of fixation. Known paintings rated aptly were characterized by a smaller number of fixations than unknown.

In contrast to the effects found in the dwell time analysis, the number of fixations differentiated people with different levels of expertise, understood both as the effect of education and the accuracy of aesthetic evaluation of paintings viewed in pairs. The interaction of expertise and familiarity of paintings revealed that only in the expert group the number of fixations on unknown paintings was significantly higher than on the known ones. Most likely, this was due to the greater need to explore unknown paintings by experts for whom the composition is an important criterion for assessing their aesthetic value. Non-experts equally often fixate their eyes on known and unknown paintings. A larger number of fixations on unknown paintings seems to be the good marker of expertise.

In turn, the interaction of the accuracy of paintings evaluation, expertise and the difference between paintings in a pair reveals that in the expert group a larger number of fixations predicts less accurate choice of the original painting than its altered version in pair, as more valuable in terms of aesthetics. The greater the difference between the original painting and its altered version in each pair the more clearly this effect. The occurrence of this effect indicates the inhomogeneity of the group of experts due to the aesthetic evaluation of the paintings viewed in pairs. If we agree that a painting with a balanced composition is more pleasant to view than its violated version, it turns out that at least some experts do not think so. What is more, their inaccurate evaluation is preceded by a significantly higher number of fixations on the compared images, than the accurate one.

Summing up the results of eye movement analyses in relation to the accuracy of paintings evaluation, we found several regularities: (1) regardless of the expertise understood as education in visual arts and familiarity of paintings the shorter the dwell time and average fixation duration the more accurate evaluation of paintings; (2) similarly, but only in the expert group the smaller the number of fixations the more appreciated is the balanced painting than its altered version; (3) regardless of the expertise, the longer the first fixation duration on known paintings the more likely is an inaccurate selection of the less-balanced painting than its original as more valuable aesthetically. The opposite is true for unknown paintings. The longer first fixation duration is preceded by an accurate selection of a painting with a more balanced composition.

The results of the presented analysis of the accuracy of paintings evaluation in pairs and eye movements of experts and non-experts lead to the conclusion that although people educated in the field of visual arts more accurately appreciate compositional values of paintings than non-experts, they do not constitute a homogeneous group. In both compared groups there are people whose high accuracy of assessments is preceded by a specific eye movement. Simplify, the accuracy of the aesthetic evaluation of the paintings viewed is the higher the more economical their visual exploration is. An expert, in the understanding of a person who more appreciates a balanced composition from the violated one, sees it much faster and with less effort, regardless of the level of education in the field of visual arts.

Both the objectives of the present study and the procedure used to present the paintings and their evaluation differed from those most commonly used in empirical aesthetics research, so the interpretation of achieved results must be done very carefully. Nevertheless, in our opinion, their value lies primarily in that they give a good point of reference for the results of the next study conducted in the paradigm proposed in this study.

## Ethics Statement

We confirm that APA ethical standards were followed in the conduct of the study. This study was carried out in accordance with the recommendations of the Ethics Committee (Institute of Psychology at the John Paul II Catholic University of Lublin, Poland) with written consent from all subjects. All subject gave informed consent in accordance with the Declaration of Helsinki. This study was approved by Research Ethics Committee of the Institute of Psychology of The John Paul II Catholic University of Lublin.

## Author Contributions

PF, IZ and PA: substantial contributions to the conception of the work and substantial contributions to the design of the work. PF, PA, NK and TJ: the acquisition, analysis, or interpretation of data for the work. PF: drafting the work. PF, IZ, PA, NK and TJ: revising the work critically for important intellectual content, final approval of the version to be published and agreement to be accountable for all aspects of the work in ensuring that questions related to the accuracy or integrity of any part of the work are appropriately investigated and resolved.

## Conflict of Interest Statement

The authors declare that the research was conducted in the absence of any commercial or financial relationships that could be construed as a potential conflict of interest.
