# Supplementary material for: Eye Movement Correlates of Expertise in Visual Arts
Source: Front Hum Neurosci. 2018 Mar 26;12:87. doi: 10.3389/fnhum.2018.00087 (PMC5879988; doi:10.3389/fnhum.2018.00087)
Supplement: Supplementary file 1 [file Data_Sheet_1.docx]

Links to paintings with a balanced composition used in the study

1. by famous artists
2. Pieter Bruegel the Elder, *The Procession to Calvary*, <https://www.wikiart.org/en/pieter-bruegel-the-elder/christ-carrying-the-cross-1564>
3. [Marc Chagall](https://www.wikiart.org/en/marc-chagall), *The Birthday*, <https://www.wikiart.org/en/marc-chagall/the-birthday-1915>
4. Jean Fouquet, *Madonna and Child. Right Panel of Diptych de Melun*, <https://www.wikiart.org/en/jean-fouquet/madonna-and-child-left-panel-of-diptych-de-melun>
5. Wassily Kandinsky, *Transverse Line*, <https://www.wikiart.org/en/wassily-kandinsky/transverse-line-1923>
6. [Boris Kustodiev](https://www.wikiart.org/en/boris-kustodiev), *The Beauty*, <https://www.wikiart.org/en/boris-kustodiev/beauty-1915>
7. Laurence Stephen Lowry, *The Lake*, <https://pl.pinterest.com/pin/264727284318610216/>
8. August Macke, *Landscape with Cows and a Camel*, <https://www.wikiart.org/en/august-macke/landscape-with-cows-and-a-camel>
9. Henri Matisse, *Pianist and Checker Players*, <https://www.wikiart.org/en/henri-matisse/pianist-and-checker-players-1924>
10. Henri Matisse, *The Joy of Life*, <https://www.wikiart.org/en/henri-matisse/the-joy-of-life-1906>
11. Henri Matisse, *Woman in Blue*, <https://www.wikiart.org/en/henri-matisse/woman-in-blue-or-the-large-blue-robe-and-mimosas-1937>
12. Joan Miro, *The Vegetable Garden with Donkey*, <https://www.wikiart.org/en/joan-miro/the-vegetable-garden-with-donkey>
13. Pablo Picasso, *The girls of Avignon*, <https://www.wikiart.org/en/pablo-picasso/the-girls-of-avignon-1907>
14. Camille Pissarro, *Red Roofs, Corner of a Village, Winter*, <https://www.wikiart.org/en/camille-pissarro/red-roofs-corner-of-a-village-winter-1877>
15. Pierre-Auguste Renoir, *Three Bathers With Crab*, <https://fineartamerica.com/featured/three-bathers-with-crab-pierre-auguste-renoir.html>
16. Henri Rousseau, The Tiger Hunt, <https://www.wikiart.org/en/henri-rousseau/the-tiger-hunt-1896>
17. Georges Seurat, *Bathers at Asnières*, <https://www.wikiart.org/en/georges-seurat/bathers-at-asni-res-1884>
18. by relatively unknown artist
19. Iwo Zaniewski, *5 Mycie głowy*, <http://www.iwozaniewski.com/img/1422>
20. Iwo Zaniewski, *Cats and Oranges*, <http://www.iwozaniewski.com/img/1403>
21. Iwo Zaniewski, *Chess in the Countryside*, <http://www.iwozaniewski.com/img/1397>
22. Iwo Zaniewski, *Coffee in the Kitchen*, <http://www.iwozaniewski.com/img/1456>
23. Iwo Zaniewski, *Four Sisters and Mummy*, <http://www.iwozaniewski.com/img/1407>
24. Iwo Zaniewski, *Mowing Grass in Africa*, <http://www.iwozaniewski.com/img/1395>
25. Iwo Zaniewski, *Seamstresses and Sea View*, <http://www.iwozaniewski.com/img/1393>
26. Iwo Zaniewski, *Woman and Two Cats*, <http://www.iwozaniewski.com/img/1398>
27. Iwo Zaniewski, *Woman Sitting on a Bed*, <http://www.iwozaniewski.com/img/1347>
